# Wearable devices for continuous monitoring of biosignals: Challenges and opportunities

**DOI:** 10.1063/5.0086935

**Published:** 2022-04-13

**Authors:** Tucker Stuart, Jessica Hanna, Philipp Gutruf

**Affiliations:** 1 Department of Biomedical Engineering, University of Arizona, Tucson, Arizona 85721, USA; 2 Department of Electrical and Computer Engineering, University of Arizona, Tucson, Arizona 85721, USA; 3 Bio5 Institute, University of Arizona, Tucson, Arizona 85721, USA; 4 Neuroscience GIDP, University of Arizona, Tucson, Arizona 85721, USA

## Abstract

The ability for wearable devices to collect high-fidelity biosignals continuously over weeks and months at a time has become an increasingly sought-after characteristic to provide advanced diagnostic and therapeutic capabilities. Wearable devices for this purpose face a multitude of challenges such as formfactors with long-term user acceptance and power supplies that enable continuous operation without requiring extensive user interaction. This review summarizes design considerations associated with these attributes and summarizes recent advances toward continuous operation with high-fidelity biosignal recording abilities. The review also provides insight into systematic barriers for these device archetypes and outlines most promising technological approaches to expand capabilities. We conclude with a summary of current developments of hardware and approaches for embedded artificial intelligence in this wearable device class, which is pivotal for next generation autonomous diagnostic, therapeutic, and assistive health tools.

## CRITERIA FOR LONG-TERM WEARABLE DEVICES

**FIG. 1.**
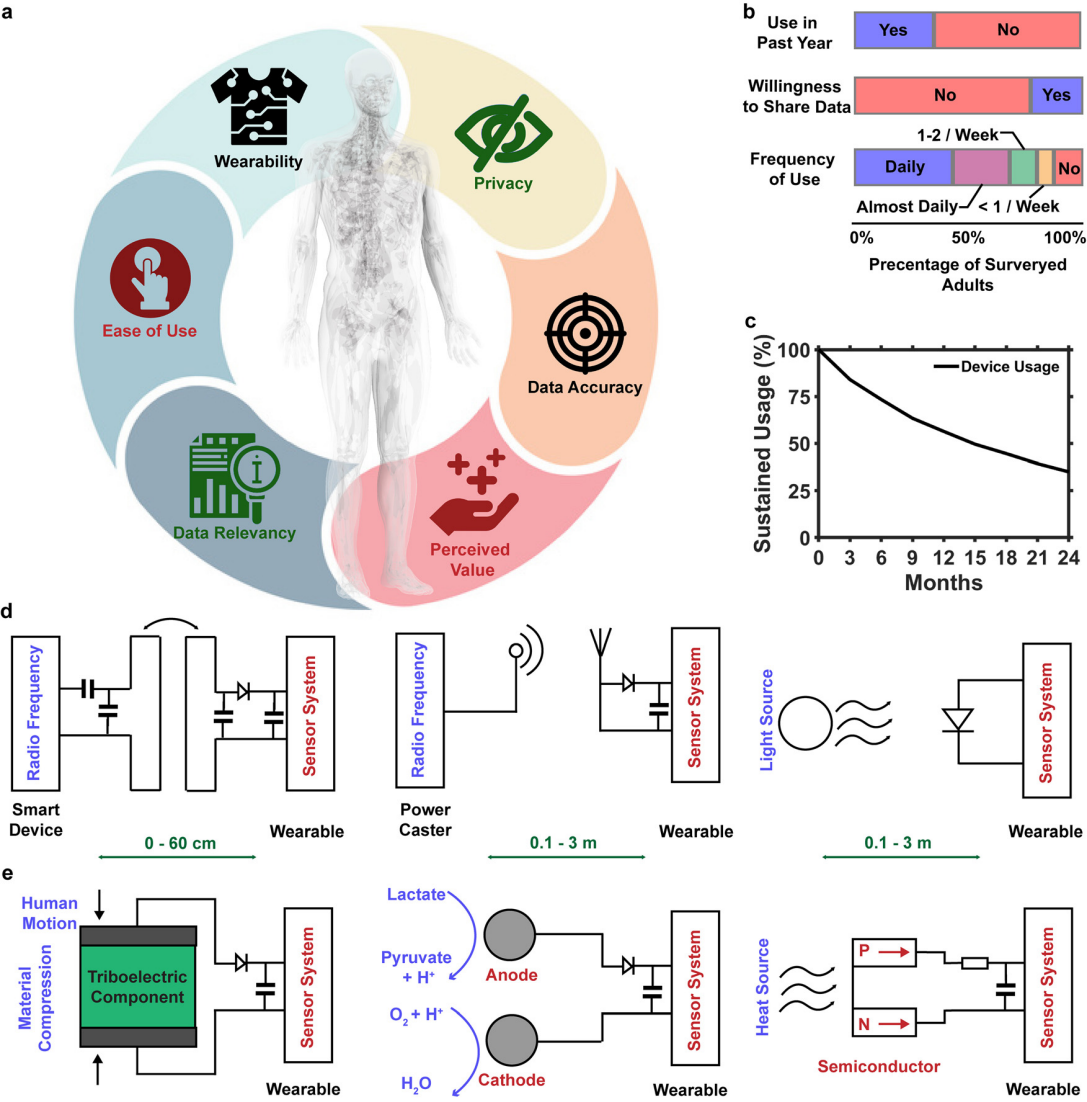
Introduction into current wearable systems: (a) illustration showing key aspects for chronic user compliance with wearable medical devices. (b) Graph showing metrics of user retention for fitness wearables.^25^ Reproduced with permission from Chandrasekaran *et al.*, J. Med. Internet Res. **22**, e22443 (2020). Copyright 2020 Authors, licensed under a Creative commons Attribution (CC BY) license. (c) Graph showing user compliance with wearable monitors over a 2-year period ledger.^27^ Reproduced with permission from D. Ledger and McCaffrey, Endeavour Partners **200**, 1 (2014). Copyright 2014 Authors, licensed under a Creative commons Attribution (CC BY) license. (d) Illustrations outlining wireless power transfer techniques' battery-free or chronic wearable devices. (e) Summary of power harvesting techniques used for autonomous wearable devices.

Wearable devices have seen an acute increase in popularity over the last 10 years.^1–7^ As the rate of proliferation of wearable systems and technological advances increases, understanding end user behaviors and factors that influence a user's decision to utilize wearable devices is an important factor to consider in the development of new device archetypes, specifically when the technologies are used as diagnostic tools. To understand the multifaceted decision process, several models have been developed to understand technology acceptance and long-term compliance. The two most popular models are the technology acceptance model^8^ and the unified theory of acceptance and use of the technology model^9^ with other works focused on expanding and refining these models.^10–23^ Key outcomes from these models are detailed in Fig. 1(a), which shows the primary factors of wearable technology systems that influence device acceptance and adoption. Among these factors, there are three primary categories, which include the following: intrinsic device hardware properties such as comfort, safety, and data relevancy and user-centered subjective factors such as perceived ease of use, perceived value, and social acceptance. When engaging in fundamental research and device design, these factors play a large role in the acceptance and adoption of new devices and, thus, must be considered early in the design process to enable impact.

The US market for wearable devices reached $24.57 billion in 2018 with a projected growth rate of 24.7% annually to $139.35 billion by 2026^24^ with nearly 30% of the population reporting using a wearable device, of which 47% using devices every day.^25^ A graph showing survey data from a sample population of 4551 participants is shown in Fig. 1(b). From this study, it was found that younger age demographics contribute more to the ownership and adoption of wearable sensing systems, whereas older users are less likely to adopt these devices (odds ratio 0.46–0.57). Additionally, it was shown that respondents who reported a high level of technological affinity were more likely to adopt wearable devices than those who were less familiar (odds ratio 1.33).^25^ These statistics demonstrate that, in general, younger and health-conscious users who are interested in fitness optimization and older populations who are focused on overall health improvement are driving market expansion, particularly accentuated during the COVID-19 pandemic.^26^

Despite widespread adoption among several subpopulations, conventional wearable devices do not meet many standards set out by acceptance models, as many devices fail to drive long-term user engagement. Figure 1(c) summarizes user engagement with wearable systems over a 2 year period.^27^ In this study, it was reported that a third of users who owned a modern activity tracking device stopped using it within 6 months of receiving it. While temporal decreases in user compliance are widely studied and depend on the formfactor of the device, users may choose to discontinue interaction with a wearable system because of perceived complication of use, a failure to integrate with existing infrastructure, discomfort, and lack of perceived value of the insight collected from wearing the device.^28–30^ These aspects of human behavior are key for long-term compliance with wearable systems, where software approaches to promote habit formation, social motivation, and goal reinforcement are important to maintain perceived value and usefulness.^31^ A barrier in practical applications is a safe and reliable biological interface. Biocompatibility and soft mechanics can reduce the risk of irritation at interfaces whereas suitable encapsulation can eliminate the risk of harmful interaction with the skin.^32^

In addition to these factors, there are additional engineering design challenges, including communication security and privacy, power consumption, and transmission reliability (including loss of real time information),^7,33^ that must be overcome to enable truly long-term, sustainable wearable devices that provide less burden to the wearer and result in higher compliance with longer wear time, all critical aspects to enable powerful health diagnostic devices.^7,32,33^

Wearable systems for noninvasive monitoring of physiological processes and biomarkers for disease detection, coupled with advances in electronic systems, materials science,^34,35^ and sensor technology^5,32^ seek to overcome these challenges. The device class is defined by wireless and battery-free operation, soft mechanics, and high-fidelity sensing capabilities to increase both user compliance and expand sensing functionality of wearable systems.^36^ Key aspects of this device class are strategies for wireless power transfer and energy harvesting, which are considered a bottleneck that drives device composition while power availability prescribes functional limitations. Commercially available devices generally utilize a form of rechargeable lithium-ion or lithium-polymer battery. Batteries impose device sizing constraints due to their large formfactor (>1 × 1 cm^2^) but are available with a wide range of capacities that can handle continuous device operation of 8–10 h. In an effort to render battery powered systems more mechanically compliant, development of stretchable cells that are able to deform and integrate seamlessly into textiles^37–39^ has proliferated; however, mechanical compliance comes at the expense of energy density resulting in larger device footprints. Wireless powering modalities for wearable systems can be categorized into two subgroups, namely, power transfer approaches that capture energy cast by external infrastructure and energy harvesting approaches that utilize existing forms of energy to power the sensor systems.^40^ Figure 1(d) details external power transfer techniques that are commonly implemented in chronic wearable device systems.

## WIRELESS POWER TRANSFER TECHNIQUES FOR BATTERY-FREE WEARABLE DEVICES

Radio frequency (RF) power transfer, which is a popular modality for wearable systems with functional operation distances of 60 cm and low tissue absorption (<20 mW/kg), makes it appealing for wearable applications directly on the skin.^41–43^ The most popular of these RF transfer modalities is near-field magnetic resonance coupling (MRC)^40^ that relies on a primary transmission coil and a resonantly coupled secondary coil that is able to provide up to 500 mW.^42^ Because of relatively robust electromagnetic makeup, technology can be united with mechanical concepts such as rigid island and stretchable interconnects to enable skin-like mechanical feel with robust operation even under large deformations,^44^ making it a popular approach for epidermal electronics. Most recent methods increase the functional distance through strategies such as near-field enabled textiles;^45,46^ MRC is generally confined to less than 1 m (Ref. 42) with high-powered transmitters and elaborate antenna schemes or sub-10 cm range with smart phones and, therefore, requires applications that either rely on sporadic readings or operation in close proximity to transmission hardware. While infrastructure is ubiquitous through near field communication (NFC) enabled phones, this solution is difficult to implement for continuous chronic operation due to the proximity requirement to the transmitter, which requires a high level of user interaction with the devices. Another method of RF power transfer focuses on the far-field regime to extend the operational distances of devices beyond 1 m and generally uses higher frequencies to sufficiently miniaturize the receiver antenna systems resulting in a harvested power of 1–100 mW depending on the distance, orientation, dielectric environment, power, and antenna gain of the transmitter and receiver.^47–49^ This allows for continuous communication strategies but requires on-body antenna structures and proximity (0.5–3 m) to power transfer hardware.^47^ Tissue adsorption (0.1–6 W/kg) and legislative limits of far field power transmission are considered the bottle neck for this technology. Strategies to improve antenna performance on the skin include implementation of 3D structures to reduce loss associated with the skin while maintaining mechanical performance.^40^ The power transfer scheme is suitable for chronic applications, and examples with operational times over weeks are demonstrated.^47^

Another power transfer technique uses photovoltaic cells (PVCs) for converting optical energy into electrical power.^50^ The PVCs in combination with near infrared and visible light sources can also be used as a power transfer scheme and are able to generate power densities of up to an estimated 1.05 mW cm^−2^ with dedicated light sources and 14.6 *μ*W cm^−2^ from ambient light sources.^51–53^ Systems can be embedded into textiles^50,54–57^ to provide power for wearable sensor devices able to detect cardiac signals.^53^ Fabrication of inorganic PVCs using processes with the ability to create ultra-small rigid island and flexible interconnect schemes to provide harvesting capabilities without compromising device mechanics. Demonstrations with this technology have shown harvesting capabilities for low-powered electronic systems; however, the current energy density of PVCs renders their wearable application limited.^53^

Energy harvesting used in wearable systems has become increasingly popular, as they do not require external power transfer infrastructure for operation. A technological overview of these power harvesting modalities is summarized in Fig. 1(e). Piezoelectric and triboelectric generators convert various mechanical energies into electrical power^58^ with power densities of 0.01–1.32 mW cm^−2^ (Refs. 59–61) and conversion efficiencies of 50%–85% (Refs. 62–64) suitable for low power wearable applications (<5 mW). While current efficiencies and power densities require large surface areas for adequate power harvesting, integration of these generators into textile components^61^ enables low sampling rate continuous applications. One downside of these modalities is the requirement for human motion for power, which renders the system ineffective during periods of sleep or rest.

Biofuel cells aimed at powering wearable devices using sweat as electrochemical power source are suitable for short term usage in epidermal electronic applications.^65–67^ Devices demonstrate a power density of 1–3.5 mW cm^−2^, sufficient to power sensing modalities and BLE SoC modules for data aggregation and communication for up to 5 h. These biofuel cells can maintain a low mechanical profile, enabling integration of soft, flexible sensor systems; however, they may suffer inconsistent performance due to difference in physiology from user to user based on differences in sweat rates and ion concentrations and have a fundamental time limit of operation associated with materials used.

Thermoelectric generators that convert difference of body temperature and ambient temperature into electrical energy to power wearable devices^68–71^ are based on the Seebeck effect^72^ that has the advantage of omission of rectification circuits; however, they only offer modest power densities and can only power applications with ultra-low power requirements (<1 mW). Devices with average power densities between 5 and 15 *μ*W cm^−2^ (Refs. 73 and 74) have been demonstrated requiring either large device size or ultra-low power operation limiting utility of this modality to measure very slow biosignals and require specialized data transmission schemes. The advantage of this harvesting method is that most of the time there is a temperature gradient that can power devices continuously.

## CONTINUOUS SENSING SYSTEMS

### Biophysical sensors

Epidermal sensors, specifically thin, battery-free solutions increase signal fidelity because of the reduction of interfacial challenges such as motion artefacts^36,75,76^ and offer access to biomarkers in biofluids (sweat and interstitial fluid), and many physiological indicators that if monitored in real time with high-fidelity expand capabilities in diagnostics and therapeutics.^40,77^ Because of the small form factor and mechanical compliance with the skin, this device class has the prospect of increasing user compliance by reducing device interaction and discomfort and increasing signal fidelity through intimate sensor interfaces resulting in a higher value of the captured biosignals^78,79^ and increasing the likelihood of long-term adoption.

**FIG. 2.**
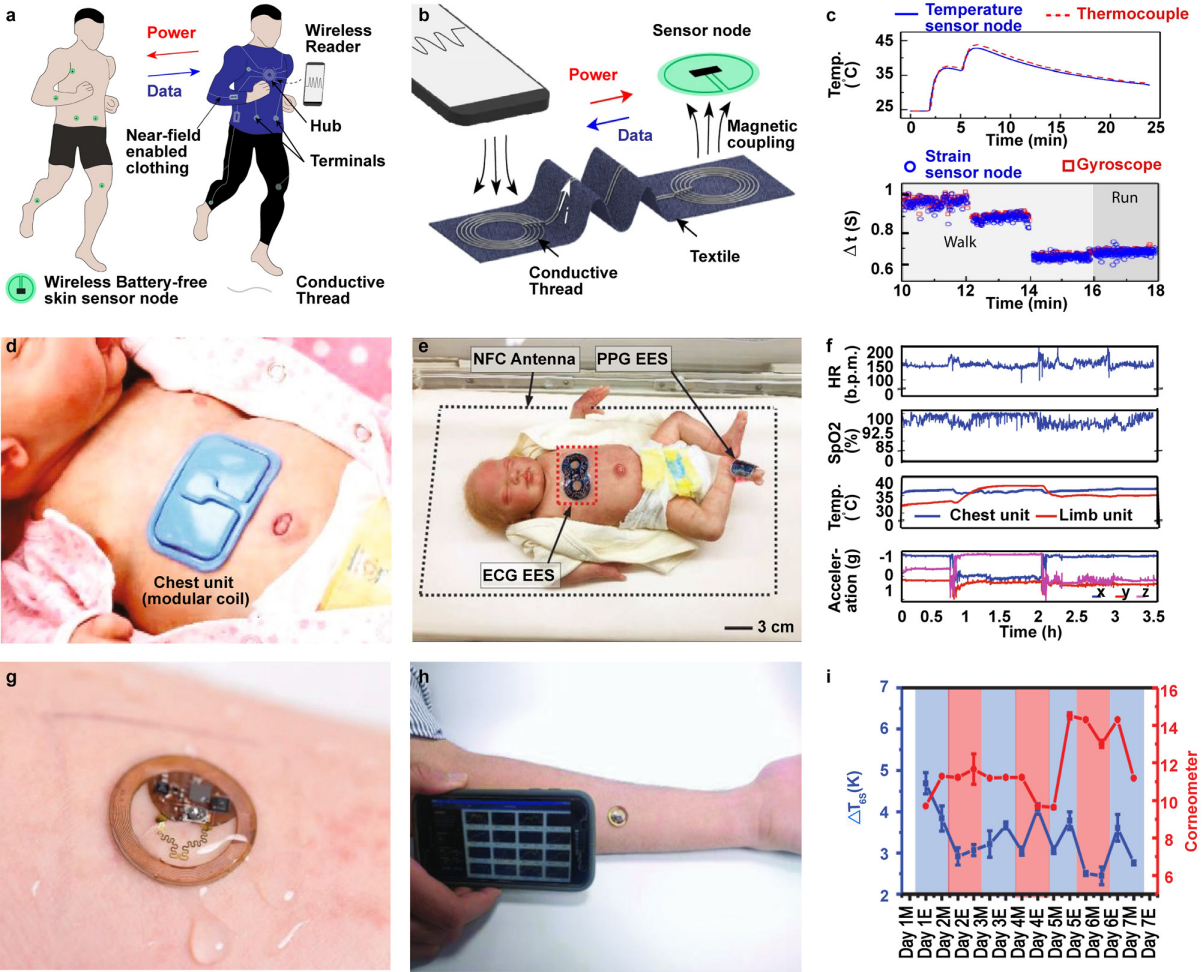
Wireless battery-free biophysical sensors. (a) Schematic illustration of wireless, battery-free multimodal wireless skin-mounted sensors powered by near-field-enabled clothing.^80^ Reproduced with permission from Lin *et al.*, Nat. Commun. **11**, 1–10 (2020). Copyright 2020 Authors, licensed under a Creative Commons Attribution (CC BY) license. (b) Illustration of conductive thread to relay power and data transfer for operation of the skin mounted sensors.^80^ Reproduced with permission from Lin *et al.*, Nat. Commun. **11**, 1–10 (2020). Copyright 2020 Authors, licensed under a Creative Commons Attribution (CC BY) license. (c) Graph showing continuous monitoring of axillary temperature and running gait during exercise using epidermal sensors, compared with the gold standard wired system.^80^ Reproduced with permission from Lin *et al.*, Nat. Commun. **11**, 1–10 (2020). Copyright 2020 Authors, licensed under a Creative Commons Attribution (CC BY) license. (d) Photographic image of the electrocardiogram (ECG) device capable of measuring HR, SpO_2_, and RR along with central-skin and peripheral-skin temperature to monitor neonatal intensive care (NICU). Reproduced with permission from Chung *et al.*, Nat. Med. **26**, 418–429 (2020). Copyright 2020 Springer Nature. (e) Photographic image showing the preterm infant in NICU bed equipped with the NFC antenna system to power the battery-free ECG and photophlethysmographgy (PPG) sensors placed on the chest and the foot of a newborn, respectively.^82^ Reproduced with permission from Chung *et al.*, Science 363, eaau0780 (2019). Copyright 2019 Authors, licensed under a Creative Commons Attribution (CC BY) license. (f) Graph showing *in vivo* data collection including HR, SpO_2_, Temp, and acceleration. Reproduced with permission from Chung *et al.*, Nat. Med. **26**, 418–429 (2020). Copyright Springer Nature. (g) Photographic image of an epidermal wireless thermal sensor (eWTS) for thermal conductivity measurements.^83^ Reproduced with permission from Krishnan *et al.*, Small **14**, 1–13 (2018). Copyright 2018 Wiley‐VCH Verlag GmbH & Co. KGaA, Weinheim. (h) Optical image showing readout from the eWTS placed on the forearm using a smartphone.^83^ Reproduced with permission from Krishnan *et al.*, Small **14**, 1–13 (2018). Copyright 2018 Wiley‐VCH Verlag GmbH & Co. KGaA, Weinheim. (i) Graph of thermal conductivity measured continuously using the eWTS for 1 week and compared to the gold standard electromagnetic method evaluating skin hydration.^83^ Reproduced with permission from Krishnan *et al.*, Small **14**, 1–13 (2018). Copyright 2018 Wiley‐VCH Verlag GmbH & Co. KGaA, Weinheim.

Lin *et al.*^80^ demonstrated a multimodal system based on skin-mounted wireless sensors powered by near-field-enabled clothing. Figure 2(a) shows the system in which the wireless sensors are attached directly onto the skin to continuously collect physiological and gait parameters. Lightweight and flexible negative temperature coefficient (NTC)-thermistor based temperature sensors and strain gauge sensors are combined with a commercial NFC chipset. These sensors are wirelessly connected to near-field-enabled clothing, which are compatible with NFC-enabled devices (such as smart phones) serving as the power supply and readout device for the distributed sensors on the body [Fig. 2(b)]. To improve the conventional NFC range of operation (<4 cm for mobile devices), near field relays were designed, which allow operation up to a meter distance. Relying on multiple inductor patterns, the authors demonstrated the ability to power up to six sensor nodes. Power transfer efficiency to each of the six sensor nodes is measured to be about 3%, which is suitable to maintain stable communication. In one experiment, a temperature sensor placed under the armpit and a strain sensor placed on the knee are used to continuously monitor axillary temperature and running gait, respectively, during exercise [Fig. 2(c)]. Because sensor nodes are readout with a device that is always carried with the user (smartphone), the likelihood of discontinuation of use because of frequent recharging of multiple gadgets is eliminated and the possibility to place devices and physiologically relevant locations increases data relevancy and fidelity offering the opportunity to increase the perceived value.

Multimodal wireless and battery-free devices for vital sign analysis based on epidermal sensors are described in Refs. 81 and 82. They are capable of measuring heart rate (HR), oxygen saturation (SpO_2_), and respiratory rate (RR) along with central-skin and peripheral-skin temperature to monitor patients in the neonatal intensive care (NICU) and pediatric intensive care unit [Fig. 2(d)]. The devices also include inertial measurement units (IMUs) to track motion and body orientation and voice biomarkers related to intonation and temporal aspects of crying, serving as a good proxy for systolic blood pressure. Battery-free operation is achieved with MRC [Fig. 2(e)]. The NICU bed is equipped with a 13.56 MHz antenna system to power the device without any direct physical contact to the newborns. A Bluetooth low energy (BLE) system-on-a-chip (SoC) enables robust wireless data transmission and real-time display of the collected data within an operational range of 10 m. Soft mechanics are engineered through an elastomeric enclosure with an inner silicone-gel liner decoupling strain from electronics and provide a soft interface to sensitive skins and highly curved anatomical features of newborns. The system was tested during and after a typical kangaroo care study, which features frequent skin-to-skin contact with parents. Figure 2(f) presents the HR, SpO_2_, skin-temperature, and the accelerometry data. The system demonstrates reliable and accurate measurements, comparable with the gold standard, hard-wired techniques, for continuous monitoring periods of up to 24 h. Motion artifacts that occur during clinical treatment, feeding, and medical imaging are reduced in scale and prevalence because of the wireless operation, mechanical stability, and wearability of the device increasing recording fidelity. The core benefit of this embodiment is the extended wearability and chronic operation without the need of device interaction that can have substantial effects on preterm infants due to very fragile skin where instruments can leave lifelong scars and the ability to improve clinical outcomes with additional skin to skin interaction with parents, which are otherwise interrupted or reduced by wired connections.^84^

Figure 2(g) presents a demonstration of epidermal electronic devices that feature a wireless thermal actuator and sensor (eWTS) with MRC and NFC for power and data transmission for thermal conductivity measurements.^83^ When placed on the skin surface, low-power thermal actuation (2 mW) is applied through a low thermal mass thin film heater in epidermal contact with the skin resulting in confined heating. Temperature measurements with the actuator that also functions as a sensing element produces time-dependent data [ΔT(t)], which is evaluated to calculate the thermal conductivity (k) and thermal diffusivity. Such measurements can provide valuable information on blood flow, skin hydration, and wound healing.^83^ Figure 2(h) shows readout from the eWTS placed on the forearm with a smartphone using NFC. The battery-free, lightweight design of these sensors enable continuous monitoring of skin temperature, skin hydration, and thermal conductivity for up to one week without interfering with daily routines such as exercising, bathing, and sleeping [Fig. 2(i)]. These characteristics serve as a demonstration for the implementation of systems that can record chronically for an extended period of time, providing valuable information about the progression of diseases and the effectiveness of the treatments. An example is the diagnosis of cerebral shunt function failure, which requires continous monitoring to provide lifesaving feedback on the implant state with location (neck) not accessible to current wearable tech.^85^

### Biochemical sensors

In addition to expanding sensing capabilities and broadening the use case as well as user compliance of biophysical sensor systems, epidermal form factors of wireless and battery-free devices enable noninvasive investigation into biochemical makers, such as lactate,^86–89^ pH,^65,86,90,91^ and glucose,^86,87,89,92,93^ without the need for transdermal device penetration, substantially reducing the barrier of device usage, expanding analysis, screening, and testing schemes.

**FIG. 3.**
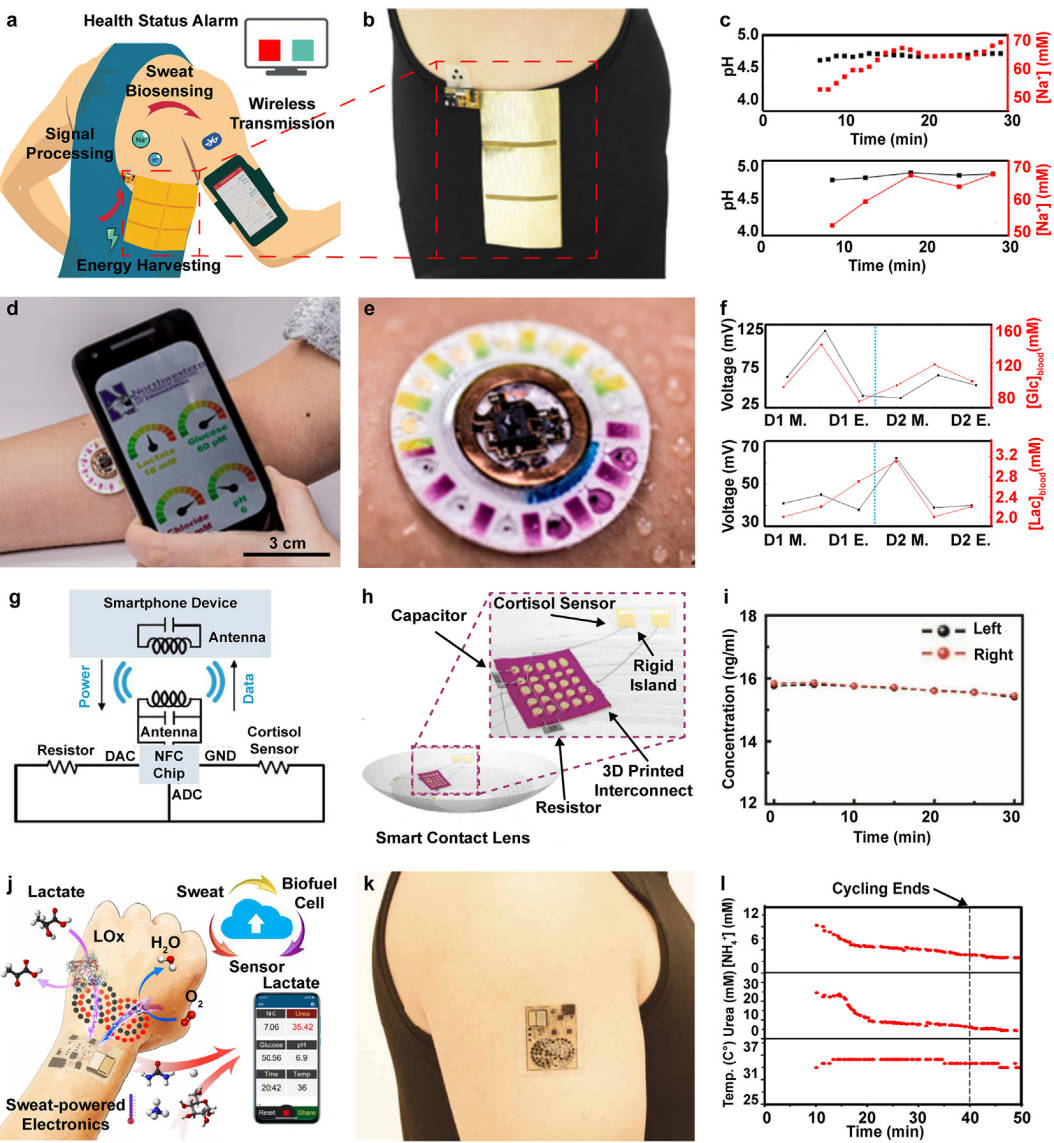
Wearable biochemical sensing devices: (a) graphical illustration showing device function and motion harvested by triboelectric nanogenerator (TENG) to power electrolyte sweat analysis.^61^ Reproduced with permission from Yu *et al.*, Sci. Adv. **6**, eaay9842 (2021). Copyright 2021 Authors, licensed under a Creative Commons Attribution (CC BY) license. (b) Image showing device applied to the subject's torso.^61^ Reproduced with permission from Yu *et al.*, Sci. Adv. **6**, eaay9842 (2021). Copyright 2021 Authors, licensed under a Creative Commons Attribution (CC BY) license. (c) Real time pH and sodium concentrations measured with the FWS^3^ device compared to the system charged with the lithium battery.^61^ Reproduced with permission from Yu *et al.*, Sci. Adv. **6**, eaay9842 (2021). Copyright 2021 Authors, licensed under a Creative Commons Attribution (CC BY) license. (d) Image of multimodal performance biomarker sweat analysis device interfacing with a smartphone for analysis readout.^86^ Reproduced with permission from Bandodkar *et al.*, Sci. Adv. **5**, eaav3294 (2019). Copyright 2019 Authors, licensed under a Creative Commons Attribution (CC BY) license. (e) Images showing device operation during perspiration.^86^ Reproduced with permission from Bandodkar *et al.*, Sci. Adv. **5**, eaav3294 (2019). Copyright 2019 Authors, licensed under a Creative Commons Attribution (CC BY) license. (f) Data correlation between seat-based glucose and lactate readings and measured values from blood level measurements.^86^ Reproduced with permission from Bandodkar *et al.*, Sci. Adv. **5**, eaav3294 (2019). Copyright 2019 Authors, licensed under a Creative Commons Attribution (CC BY) license. (g) Schematic illustration showing the electrical device operation principle of contact lens based electrochemical sensing of tear fluid.^94^ Reproduced with permission from Ku *et al.*, Sci. Adv. **6**, eabb2891 (2020). Copyright 2020 Authors, licensed under a Creative Commons Attribution (CC BY) license. (h) Illustration displaying device and sensor composition integrated in contact.^94^ Reproduced with permission from Ku *et al.*, Sci. Adv. **6**, eabb2891 (2020). Copyright 2020 Authors, licensed under a Creative Commons Attribution (CC BY) license. (I) *In vivo* cortisol concentrations collected simultaneously from both eyes of a subject.^94^ Reproduced with permission from Ku *et al.*, Sci. Adv. **6**, eabb2891 (2020). Copyright 2020 Authors, licensed under a Creative Commons Attribution (CC BY) license. (j) Illustration of biofuel-powered energy harvesting from perspiration to power electrochemical analysis of sweat. Reproduced with permission from Yu *et al.*, Sci. Robot. **5**, 1–14 (2020). Copyright 2020 AAAS. (k) Image of the device located on the subject's arm. Reproduced with permission from Yu *et al.*, Sci. Robot. **5**, 1–14 (2020). Copyright 2020 AAAS. (l) Real-time data collection of urea and NH_4_^+^ concentrations from a forehead mounted device. Reproduced with permission from Yu *et al.*, Sci. Robot. **5**, 1–14 (2020). Copyright 2020 AAAS.

Figure 3(a) shows an illustration of such a device, enabling continuous sweat analysis^61^ and utilizing human motion through a free standing triboelectric nanogenerator (FTENG) [shown in Fig. 3(b)], which can convert the mechanical energy of human motion into electrical energy to power sensing and communication electronics.^58,95–97^ The sensing component of the device features a biosensor array with on-body, laser-patterned microfluidics channel for sweat-based pH and sodium concentration detection. This biosensor array features a dynamic range of pH (4–8) and sodium concentrations (12.5–200 mM). System performance for this study was evaluated during 30-minute durations of the exercise against a battery-powered system with high compliance between the two devices. [Data are presented in Fig. 3(c).] For applications with slow physiological processes such as changes in sweat composition, a high sampling rate is not necessary, making the FTENG powered system a viable alternative to electrochemical power supplies and providing adequate power supply for device function during exercise.

Among other metabolites of interest, metabolic byproducts, such as glucose and lactate, are important biomarkers in many disease paradigms and human performance analysis and are essential in understanding underlying metabolic processes. While many of these biomarkers are present in biofluids such as blood, they traditionally require transdermal probing for accurate recording, increasing the barrier of usage significantly and making noninvasive alternatives compelling to develop. One device that provides exemplary monitoring capabilities with multimodal sensing capabilities is featured in Fig. 3(d).^86^ The device presented in this work utilizes adhesive application to the epidermis to collect sweat via epifluidics, a soft microfluidic wearable designed to capture sweat and route it to analysis chambers that house colorimetric, volumetric, and electrochemical analytes. The device utilizes a multimodal approach for its sensing functionality: a disposable patch for collection and colorimetric analysis of sweat, and a reusable electronics module for electrochemical detection of sweat-based glucose and lactate. The device, shown in Fig. 3(e), utilizes magnets to attach the components together. NFC is used to enable digital and continuous readout of the biofuel cell-based glucose and lactate sensors. This allows the user to utilize a smartphone device for readout while launching an app to trigger photographic colorimetric readout of ion concentrations and sweat rate. Figure 3(f) shows a comparison between the sensor output of sweat-based lactate and glucose concentrations to blood measurements over a 2-day period. In this study, measurements were taken during periods of fasting, right after a meal, and in the evening. In the plots, spikes in glucose concentrations after meals are observed in both the blood and sweat measurements, while trends in lactate concentrations were consistent throughout the trial. The reusable, low-cost nature of these devices provides a favorable alternative to currently invasive monitoring.

Integration of battery-free electronics into contact lenses offers direct access to tear fluid, which is rich in biomarkers that can be harnessed for diagnostic purposes.^92,94,98,99^ Device can also be made chronically wearable because there is little cell turnover. One such device is outlined in Fig. 3(g) and operates using NFC powering and communication for the readout of tear-based cortisol concentrations.^94^ Cortisol is a biomarker for endocrine disorders such as Cushing syndrome, as well as an early precursor for neurological disorders such as stress and Alzheimer's disease.^100–103^ Cortisol levels can vary widely from patient to patient and change continuously, making continuous monitoring important in identifying changes in physiology that may indicate the disease state. The device shown in Fig. 3(h) features a graphene-based sensor that is functionalized with cortisol monoclonal antibodies (C-Mab), which act as a transducer to convert interaction between cortisol and C-Mab into changes in resistance that can be detected by the analog front end of the NFC chip. The sensor features a limit of detection of 10 pg/ml, well below the cortisol concentration in tears (1–40 ng/ml) with a sensitivity of 1.84 ng/ml. Results from *in vivo* testing is shown in Fig. 3(i) from both the right and left eye of a subject at 5-min intervals during a 30-min collection period. Both devices provide similar readouts for cortisol concentrations, indicating little sensor to sensor variance.

NFC-based communication and powering for wearable systems, while having robust pre-existing infrastructure and development, are unfavorable for many long-term wearable systems as they require close proximity to a transmission device for powering and data readout.^104^ Utilization of biofuel cells to convert sweat to usable electronic energy provides a potential avenue for applications that require operation outside of existing infrastructure. The device illustrated in Fig. 3(j) shows one such system^66^ capable of measuring key metabolic biomarkers. The device shown in Fig. 3(k) is comprised of a biofuel cell array with an energy density of 3.6 mW cm^−2^ in untreated human sweat samples and good long-term stability over 2000 cycles. Collection of urea and NH_4_^+^ concentrations were carried out using electrochemical patches based on ion-selective electrodes with enzymatic layers and can measure concentrations of 2.5–40 mM for both metabolites. Validation of device operation was carried out during a stationary biking exercise with data presented in Fig. 3(l). As expected, a decrease in urea and NH_4_^+^ concentrations is observed during the exercise period, which then stabilized after conclusion of cycling.

Wireless and battery-free biochemical sensors highlight an area of development with potentially high impact if device architectures allow for chronic recording capability that would otherwise require blood draw and laboratory testing, which is often a barrier to preventative measures that results in late diagnosis^105^ and limited metrics for continuous disease management and care.^106,107^

## CURRENT LIMITATIONS OF WEARABLE DEVICES

The devices discussed in this review feature advances in materials science,^5,32,44,108,117^ electronics,^7,109–114^ and wireless power transfer^115–121^ to enable wireless and battery-free device formfactors with enhanced sensing capabilities to extract high fidelity biosignals. The goal of these devices is often to expand sensing functionality while improving user comfort and compliance with the hope to increase device acceptance. While for some applications such as screening and some diagnostic applications operating times are well below 1 week, where current schemes offer significant advantages over contemporary approaches, chronic operation (greater than 2 weeks at a time) has yet to be achieved. The significance of this chronic operation is progression toward the goal of continuous multimodal monitoring to enable digital medicine,^122,123^ namely, early automated diagnosis,^124–128^ personalized therapeutics,^129–131^ and individualized chronic care approaches.^132^

**FIG. 4.**
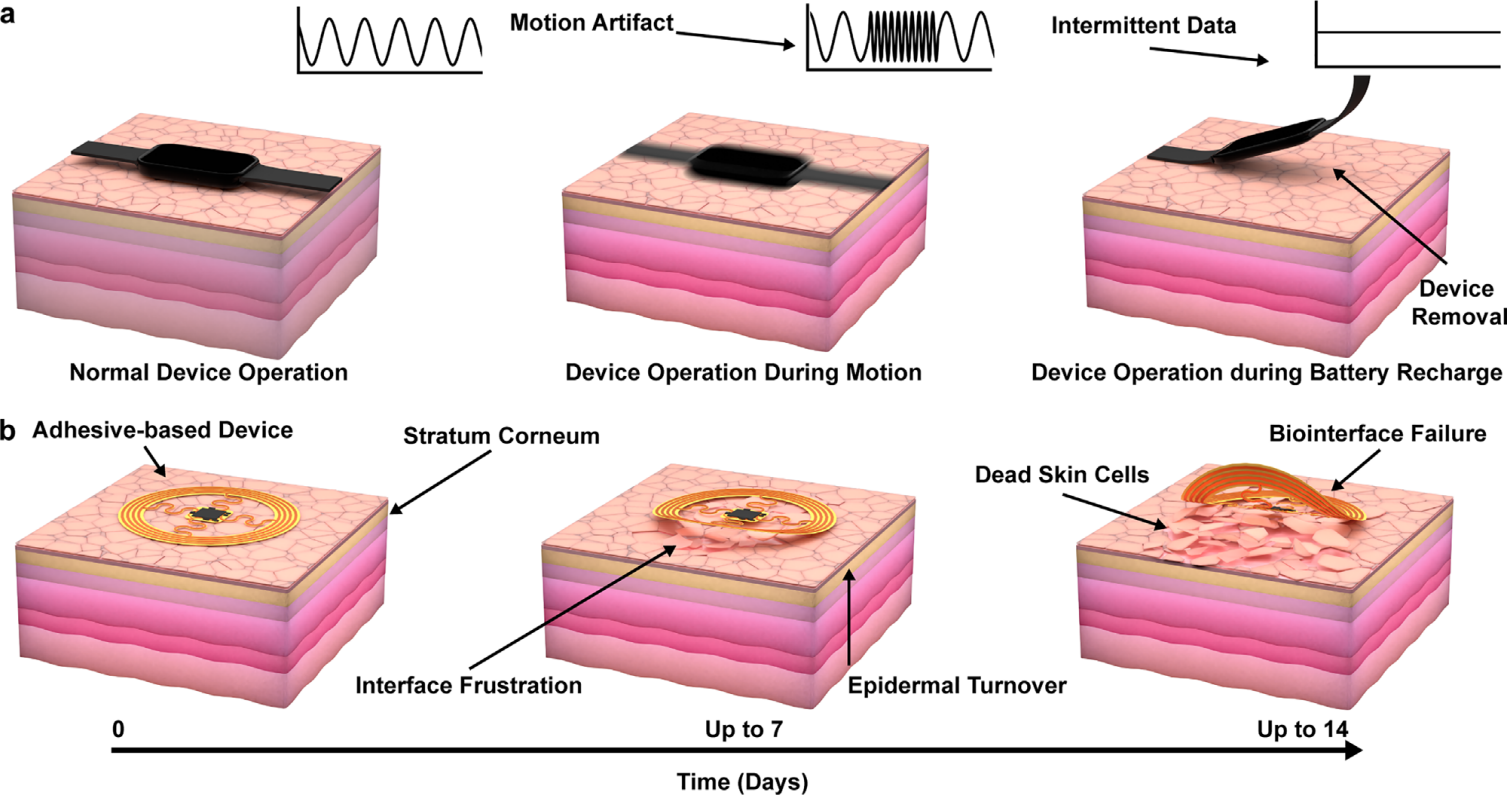
Limitations of current wearable devices for biosensing applications. (a) Illustration showing conventional wrist worn wearable systems during operation. (b) Illustration of wearables utilizing adhesive interfaces during operation with key limitations in epidermal turnover.

Battery-powered wearable systems, such as smartwatches or fitness trackers, offer a low barrier of entry to the consumer market, however, they incur penalties in sensing quality, useability, and user compliance.^133,134^ These issues are illustrated in Fig. 4(a). During normal device operation, there are frequent periods of biosignal interruption due to motion artifacts associated with the mass of the battery and formfactors that result in lack of conformality to the sensing target. Often, sensors are housed in large, rigid containers with masses ranging from 30 to 350 g, where rapid movement can disrupt the sensing interface.^135^ Utilization of a finite batteries requires the need for daily charging periods where devices must be removed, resulting in prolonged periods of data loss and increased chances of user noncompliance. This is compounded by device removal during certain activities such as sleep or hot weather due to user discomfort associated with the large device formfactor. Additionally, sensing regions are constrained to a small surface, typically on a peripheral appendage, which limits the sensor functionality and fidelity while decreasing physiological relavancy.^136,137^ Combined with other subjective requirements for user acceptance, such as discreet wearability and perceived value, result in underperforming user compliance, even despite initial acceptance success. These disruptions result in unrecorded biosignals that may hold key insight into underlying changes in subtle physiological parameters.

Current behavioral research suggests that ease of use, among other factors, played a role in low user acceptance of wearable systems over time.^19,31,138^ Studies also found large portions of daily data are low fidelity, which limits the scope and data aggregation capabilities restricting wholistic view of physiological phenomena.

Epidermal devices that provide skin-like mechanics, low dimensional profiles, and wireless power transfer functionalities enable high-fidelity biosignal acquisition by removal of weight associated with large battery-powered supplies with the ability to be deployed virtually anywhere on the body. Devices of these form factors, however, are insufficient to meet the demands of chronic data recording due to their inconsistency of power delivery to the system and their use of adhesive-based application for epidermal contact. The shortcomings of these devices are summarized in Fig. 4(b). Epidermal turnover, characterized by the cell renewal of the stratum corneum, is a barrier to longevity of such a device, as it frustrates the sensing interface and renders the adhesive bond to the skin nonfunctional, prohibiting recording or stimulation beyond 7–14 days depending on the skin renewal rate and location of the device.^139^

This physiology confines the use of adhesive based devices to acute applications or requires frequent renewal of the adhesive, which, for example, in a hospital setting is completely acceptable; however, it is less appealing for at-home diagnostic applications. Additionally in some cases, the utilization of strong adhesives may bar users from utilizing the device due to skin sensitivity,^140^ while the requirement for periodic replacement adds further burden to the user, which may affect long-term compliance and interrupt period of data collection.

## OPPORTUNITIES FOR LONG-TERM WEARABLE DEVICES

**FIG. 5.**
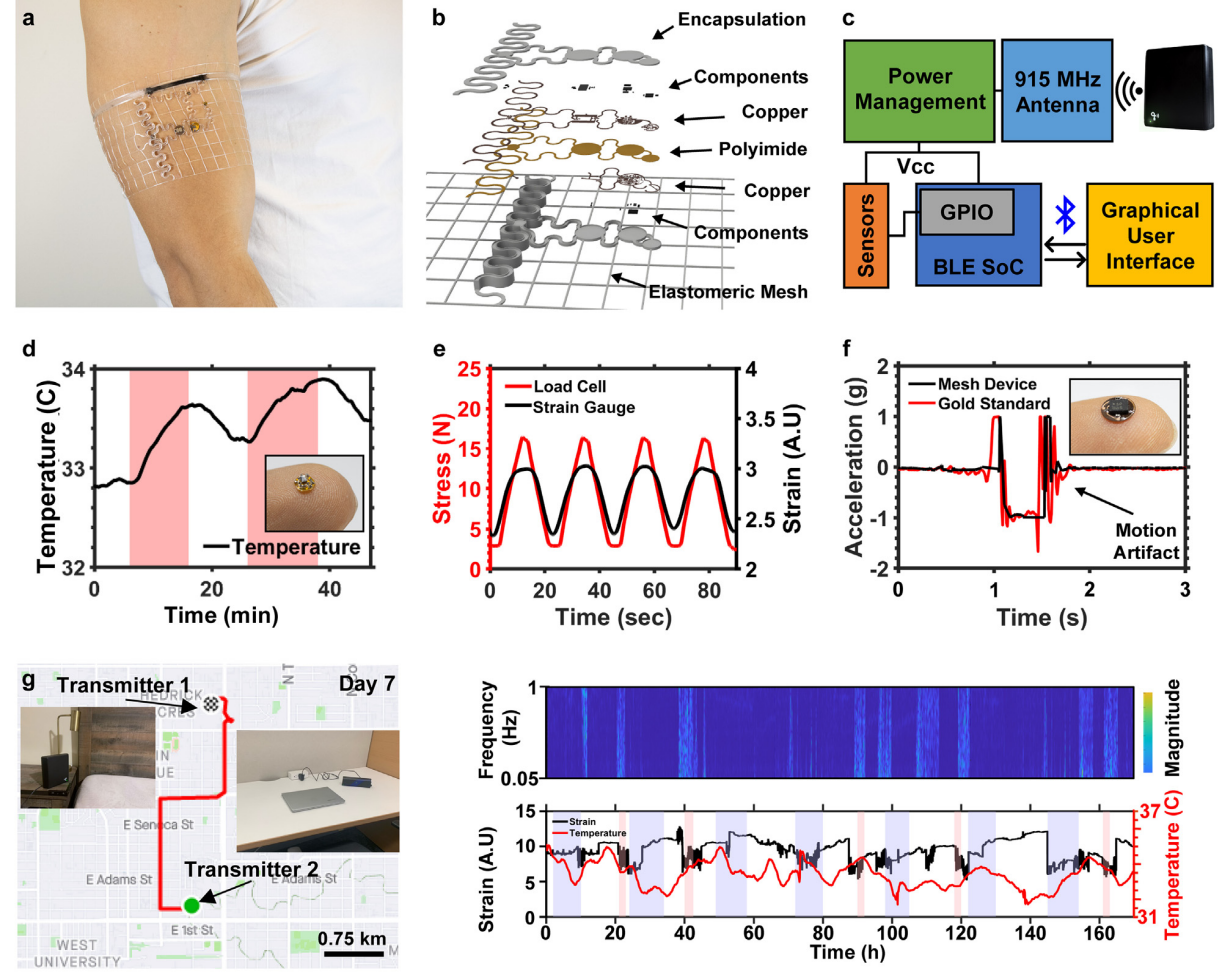
Long-term wearable sensing devices: (a) image showing biosymbiotic device for the biceps with inset illustrating the personalized digital design and fabrication process. (b) Layered schematic of biosymbiotic devices. (c) Functional diagram of the operating principle. (d) Realtime recording of body temperature collected from the axilla region during sitting (white) and walking (red). (e) Graph showing cyclic response of the 3D printed strain gauge during 16 N of applied stress (red) and corresponding change in resistance (black). (f) Graph showing lower leg acceleration of the biosymbiotic device (black) and gold standard (red) during a stationary jump. (g) Continuous data and sample global positioning system (GPS) mapping from a 7-day recording of body temperature and muscular strain captured by a biosymbiotic device worn on the proximal bicep. Adapted from Tucker *et al.*, Sci. Adv. **7**, eabj3269 (2021). Copyright 2021 Authors, licensed under a Creative Commons Attribution (CC BY) license.^47^

To overcome user compliance challenges while providing long-term uninterrupted streams of high-fidelity data, a new class of wearable systems is needed. These devices must provide a robust platform that features body-like mechanical properties with high-fidelity sensing capabilities, long-range power transfer capabilities that enable reliable device operation without constraints of short-distance power transfer techniques, and miniaturized electronics that provide the uncompromised system function with low-profile and small footprint, in a package that enhances the user experience to the point that perceived value from the data far outweighs the burden of wearing a device. An example of such a device class called a biosymbiotic device is featured in Fig. 5(a).^47^ Devices are designed utilizing 3D scanning techniques^141–145^ to collect the users' topological data to tailor device mechanics and sensing capabilities directly to applications and need of the individual user. The device is fabricated using a 3D printed thermoplastic polyurethane (TPU) material that is digitally manufactured tailored to the users unique physiology.^47^ Embedded in the mesh are electronic systems [see Fig. 5(b)], which provide multimodal function while maintaining a low mechanical profile. Topological control offered by 3D printing allows for tuning of discrete and bulk mechanics, which enables epidermis like device mechanics and facilitates design of electromagnetics to optimize energy harvesting capabilities. A block diagram detailing the device operation principle is shown in Fig. 5(c). The device utilizes RF power transfer at 915 MHz to recharge the system wirelessly. Power harvested from the on-body antenna is directed to a power management system, which stores excess energy in a supercapacitor or miniaturized battery. The stored energy is used to power a BLE SoC, which collects information from peripheral sensors and digitally communicates data to a user interface for data analysis and storage.

The conformality and mechanical profile of the device enables multimodal sensing capabilities matching or exceeding and expanding the current gold standard in battery-powered wearable systems. This has been demonstrated with several sensing capabilities including sub-millikelvin thermography, muscular deformation, and high-precision inertial measurements. Figure 5(d) shows the sub millikelvin resolution temperature sensor realized in a 2.5 mm node (shown in the inset, balanced on finger). The low thermal mass of the device enables fast response to small changes in the surface body temperature that enables detection of physiological changes, such as the difference in sitting and moderately paced walking, with axilla skin temperature change of less than 1 °C per event. Fidelity shown with this device class can provide valuable insight into acute or subtle variations indicating onset of disease.^91,146–152^

The circumferential fit and use of 3D printing also enables unique sensing capabilities that are currently not possible with conventional devices. One such modality is presented in Fig. 5(e), where a 3D printed, conductive TPU is used as a strain gauge to measure muscular deformation during activity. User specific placement and design can also increase signal fidelity. The graph in Fig. 5(e) shows the cyclic response of the strain gauge compared to input strain suitable for quantification of muscular deformation. The imperceptible weight (<500 mg) of the device also allows for highly accurate inertial measurements [see Fig. 5(f)]. A high-performance (IMU) realized in a 4 mm diameter node is compared to the gold standard, battery-powered system that is used for in-clinic assessment. The graph shows a subject performing a jump, in which the gold standard device shows motion artifacts due to device bulk inertia while the conformal system shows no motion artifacts. Removal of motion artifacts both increase data relevancy and reduce gaps in usable data, yielding accurate, long-term data collection capabilities.^153,154^

Advances of biosymbiotic electronics enable continuous streams of high-fidelity biosignals due to the ability for the system to wirelessly recharge without user interaction with charging infrastructure. This allows for previously untapped insight into several physiological processes that require continuous examination over extended periods demonstrated in Fig. 5(g). In the experiment, wireless power transmitters are set up in locations with high temporal occupancy to facilitate wireless power transfer. The plots demonstrate continuous acquisition of bicep deformation and core body temperature over a week-long study. Clear peaks of muscular deformation frequency and circadian rhythm can be observed correlating with logged activity and periods of rest. Data collected in this method will provide users with insight that is unattainable with the current standard in wearable technology sensing.

## OPPORTUNITIES FOR ARTIFICIAL INTELLIGENCE AS MEANS OF ON-SENSOR AUTOMATED DATA PROCESSING

**FIG. 6.**
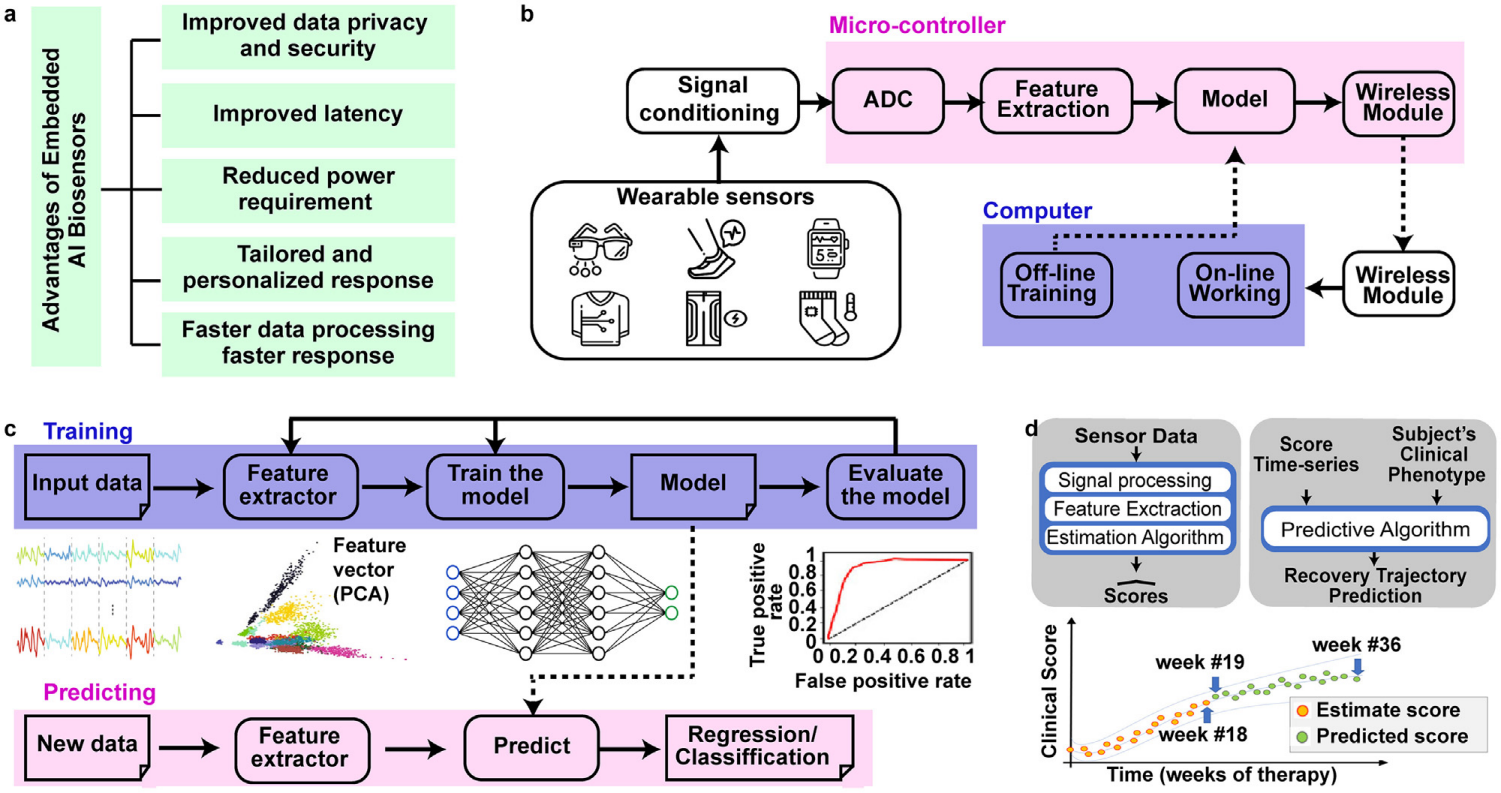
Embedded artificial intelligence. (a) Advantages of embedded AI biosensors. (b) Block diagram of functional components of wearable devices with embedded AI. (c) Workflow for training and testing Machine learning models. (d) Graph showing the motor recovery trajectory using wearable smart biosensors.^159^ Reproduced with permission from Adans-Dester *et al.*, npj Digital Med. **3**, 1–10 (2020). Copyright 2020 Authors, licensed under a Creative Commons Attribution (CC BY) license.

Wearable sensors can provide continuous, noninvasive, real-time monitoring of a wide range of physiological parameters. However, most of the available sensors are considered data collectors and/or alerting units with limited processing capabilities. Artificial intelligence (AI) offers a new path to turn biosensors into smart computational units, capable of making autonomous decisions and on-site detection/diagnosis, which is especially relevant to continuously recording devices such as biosymbiotic electronics. Local computation presents many opportunities in terms of data safety, latency,^155,156^ power consumption,^157,158^ personalized treatments, and data privacy and security^155,156^ [Fig. 6(a)]. Currently, wearable sensors are typically connected to another master device, usually a smartphone, for analytic purposes. This increases the risk compromised data safety and confidentiality because raw data are sent over the air. Processing the collected data on-device reduces privacy concerns and other cybersecurity threats. Additionally, sending data to the smartphone/cloud causes undesired delay and generally is inefficient due to relatively large power consumption of wireless radios. Conducting on-device machine learning (ML) and real-time execution reduces the data transmission rate, data latencies, and has the potential to reduce power consumption, especially for chronic recording with high sampling rates.

A wearable AI system usually consists of wearable sensors, analog front-end circuit responsible for amplifying, filtering, and signal conditioning the collected raw data from the sensors and a microcontroller used as the central processor, as shown in Fig. 6(b). With the emergence of on chip ML hardware in small packages^156^ and with low power consumption (TinyML paradigm), the AI algorithm can be executed on the system on a chip where event detection, feature extraction, and prediction can be computed. The regression/classification results can be sent wirelessly to a computer or smartphone for notification, offering tremendous opportunity for the device classes discussed in this review.

To train and test the regression/classification model, the following workflow, shown in Fig. 6(c), must be implemented for each sensor. Preprocessing of raw sensor data is usually required. Data compression, baseline drift removal, normalization, transformations, and other system-specific preprocessing methods are included. The processed data are then divided into training (approximately 60%), validation (20%), and testing (20%) sets.^160^ The training set is used to create the model and define the best algorithm hyperparameters. The validation set is used to tune these hyperparameters, and the testing set is utilized to evaluate the performance of the model. Prior to creating a model, feature extraction techniques are utilized to minimize the number of input variables. The reduction of the number of input variable results in reducing the computational cost of modeling and, in some situations, enhancing the model's performance.^160^ For wearable systems that are limited in computational resources, the model training is performed off-line first. The trained model is then embedded inside the microcontroller. On the microcontroller, the model is used to perform inference, making the wearable system a platform capable of making autonomous decisions.

Observing, classifying, and assessing human gestures/movements,^161–163^ predicting species or concentration of analytes, detecting arrhythmias,^164^ and enabling precision rehabilitation interventions are enabled with AI. An example of these capabilities is given by Dester *et al.*^159^ that use wearable accelerometer sensors placed on the arm, forearm, and fingers to assess the upper-limb motor function recovery after traumatic brain injury (TBI) or stroke. The data were collected from 37 patients (16 stroke survivor and 21 TBI survivor) during the performance of functional motor tasks. The data are then processed using ML techniques to estimate clinical scores measuring motor impairments and movement quality [e.g., the upper limb Fugl-Meyer assessment and the Functional Ability Scale]. Figure 6(d) shows the data collected from a patient during 36-week intervention. After 18 weeks, clinical scores are estimated using a ML algorithm. These scores characterize the motor recovery trajectory seen in response to the intervention during these 18 weeks [orange circles in Fig. 6(d)]. To predict the patient response to this intervention for the upcoming 18 weeks [green circles in Fig. 6(d)], the estimated clinical scores are used in conjunction with the clinical phenotype as inputs to the Gaussian process regression model (ML algorithm). Rehabilitation professionals can utilize these data to see if the patient is responding appropriately to the current intervention or if the intervention plan needs to be adjusted, hence providing patient-specific therapies highlighting the potential of AI in aiding diagnosis and therapy. It is important to note that the AI analysis was performed offline and not on the device; however, with suitable wearable and remote platforms, completely autonomous operation is viable with the latest generation of SoC.

## DISCUSSION

Wearable medical devices for the extraction of high-fidelity biosignals are pivotal to the realization of digital medicine, where technology integrates with the human body and utilizes continuous data collection and advances in AI for treatment, diagnosis, and ultimately prevention of disease. Requirements for this paradigm include wearable devices that can be worn continuously to provide uninterrupted steams of clinical grade biosignals, while AI is deployed to identify trends and markers, which may indicate disease onset or changes in underlying physiology. The development of AI has greatly outpaced wearable technology for the realization of this goal. Current wrist band type wearable devices are inadequate due to their low-fidelity sensing capabilities and challenges for long-term wearability, which include both aspects of formfactors and issues with long-term user acceptance and compliance.

The emergence of chronically wearable wireless and battery-free devices provides an appealing alternative to standard wearable devices, as they have improved sensing fidelity and expand capabilities towards biosignals that previously required clinic-based wired setups. Additionally, materials and concepts used for this device class improve long-term wearability through reduction of bulk and introduction of overall soft mechanics. Despite this, many demonstrations have been limited to short-term and specific applications with day long duration due to natural physiological processes such as epidermal turnover. Further work geared toward demonstrations of chronically wearable devices in dynamic settings with high-fidelity sensing capabilities is needed. Successful implementation of on-board AI capabilities on these devices will unlock the full potential with broad applications among various disease archetypes. Specifically, these capabilities can improve diagnosis and monitoring of chronic diseases such as aging related frailty,^165^ chronic obstructive pulmonary disease,^166^ and mental health disorders.^167,168^ The development of these devices must also keep user acceptance models in mind to ensure successful translation and deployment in a large population cross section.

## Data Availability

Data sharing is not applicable to this article as no new data were created or analyzed in this study.
